# Estimating epidemiological parameters from experiments in vector access to host plants, the method of matching gradients

**DOI:** 10.1371/journal.pcbi.1007724

**Published:** 2020-03-16

**Authors:** Ruairí Donnelly, Geofrey W. Sikazwe, Christopher A. Gilligan

**Affiliations:** Department of Plant Sciences, University of Cambridge, Cambridge, United Kingdom; University of Sussex, UNITED KINGDOM

## Abstract

Estimation of pathogenic life-history values, for instance the duration a pathogen is retained in an insect vector (i.e., retention period) is of particular importance for understanding plant disease epidemiology. How can we extract values for these epidemiological parameters from conventional small-scale laboratory experiments in which transmission success is measured in relation to durations of vector access to host plants? We provide a solution to this problem by deriving formulae for the empirical curves that these experiments produce, called access period response curves (i.e., transmission success vs access period). We do this by writing simple equations for the fundamental life-cycle components of insect vectors in the laboratory. We then infer values of epidemiological parameters by matching the theoretical and empirical gradients of access period response curves. Using the example of *Cassava brown streak virus* (CBSV), which has emerged in sub-Saharan Africa and now threatens regional food security, we illustrate the method of matching gradients. We show how applying the method to published data produces a new understanding of CBSV through the inference of retention period, acquisition period and inoculation period parameters. We found that CBSV is retained for a far shorter duration in its insect vector (*Bemisia tabaci* whitefly) than had previously been assumed. Our results shed light on a number of critical factors that may be responsible for the transition of CBSV from sub- to super-threshold *R*_0_ in sub-Saharan Africa. The method is applicable to plant pathogens in general, to supply epidemiological parameter estimates that are crucial for practical management of epidemics and prediction of pandemic risk.

## Author summary

*Cassava brown streak virus* (CBSV), which was confined to coastal East Africa and the shores of Lake Malawi, has rapidly expanded its geographic range and now threatens regional food security. A recent laboratory experiment, in which vector access to plants infected with CBSV is varied, has provided plausible ranges for epidemiological parameters such as pathogen retention period (i.e., the duration that the pathogen is retained by the vector). In this work we introduce a new computational and mathematical framework for estimating epidemiological parameter values, instead of plausible ranges, from experiments in vector access to host plants. Since long distance carriage of insect vectors occurs within atmospheric air flows, the duration that the vector retains the pathogen (i.e., retention period) indirectly limits the scale of epidemics. Using our methods, we found that CBSV is retained for a far shorter duration than had previously been assumed. Our methods can be applied to the many experiments in vector access to host plants that have been conducted for numerous arthropod-transmitted plant pathogens across a range of genera. The ensuing epidemiological parameter estimates can be used in landscape computer simulations to predict epidemic risk and the prospects for control.

## Introduction

Estimation of pathogenic life-history values such as retention period for a virus in an insect vector, is of particular importance for understanding and managing plant disease for a range of arthropod-transmitted plant pathogens [1, 2]. Knowledge of parameter values such as retention period are conventionally derived from a set of standard plant pathology experiments, which we refer to here as access period experiments. These experiments yield empirical response curves for success in virus transmission as a function of the duration of time that vectors are given access to individual host plants. Since long distance carriage of insect vectors occurs within atmospheric air flows, the duration that the vector retains the pathogen (i.e., retention period) indirectly limits the distance of virus dispersal [3, 4]. Precise knowledge of epidemiological parameters and retention period in particular, is therefore key to predicting pandemic risk. There is a pressing need for methods that can infer epidemiological parameters from the limited data that access period experiments produce. Recently, *Cassava brown streak virus* (CBSV), a whitefly-transmitted *Ipomovirus*, has emerged as a serious threat to cassava production in sub-Saharan Africa [5]. Because of a small, highly mutable genome, single stranded RNA viruses like CBSV pose a continual risk as a source of emergent plant pathogens [6]. In this paper, we outline a method for extracting estimates for epidemiological parameters such as retention period, and we apply the method to derive estimates for CBSV.

**Fig 1:**
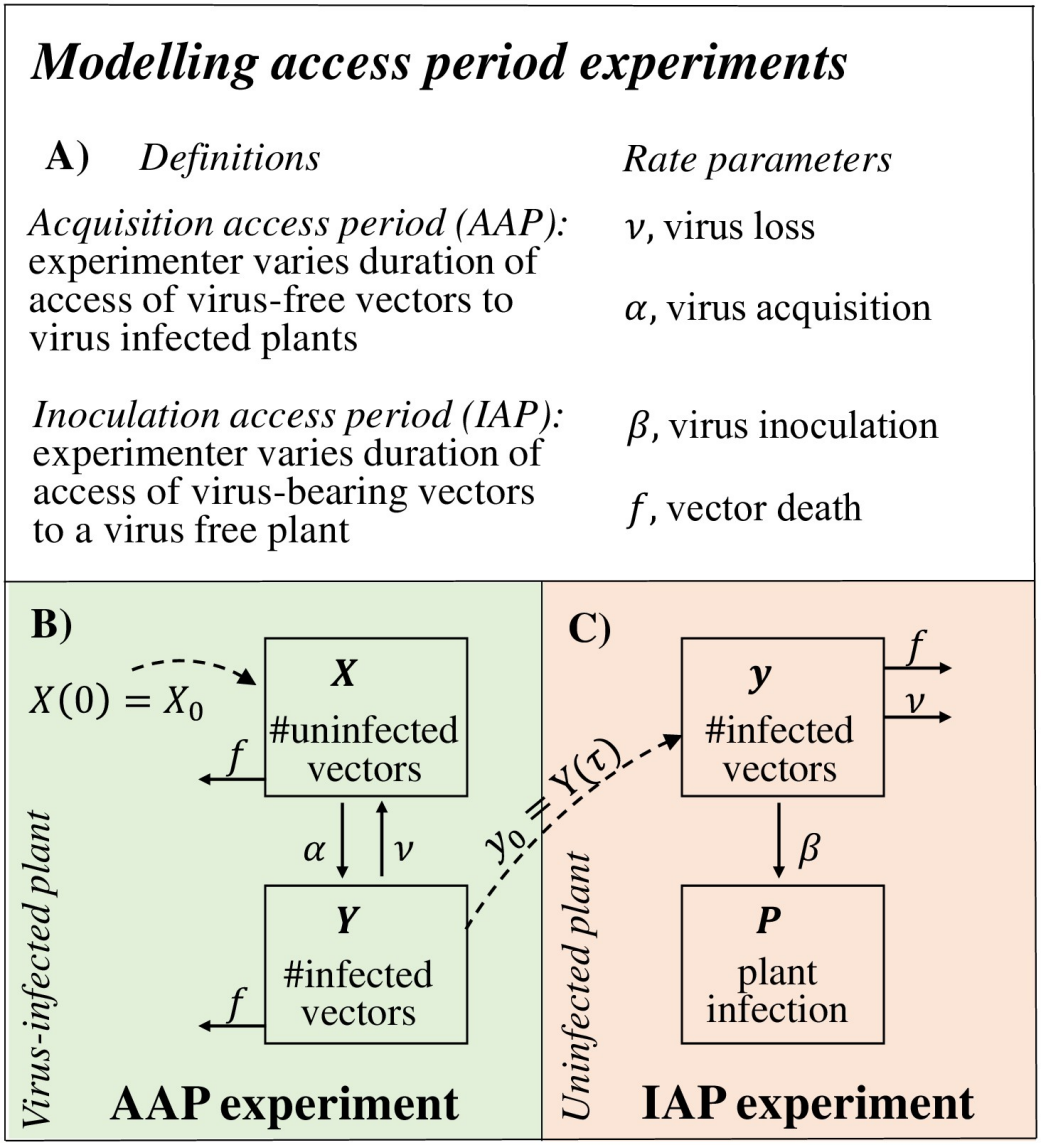
Key definitions (A) and a biological description of access period experiments (B-C). Flow charts of B: the acquisition access component, and, of C: the inoculation access component, of access period assays.

Access period experiments have been conducted for numerous plant viruses across a range of genera, from *Ipomovirus* [1] and *Begomovirus* [7] to the recently discovered *Torradovirus* genus [8]. In access period experiments, insect-borne pathogen transmission is reproduced in controlled laboratory settings, and the transmission success is recorded as the duration of exposure to the pathogen is varied. Two broad types of experiment are conducted: in the first, the experimenter varies the duration of access given to pathogen-free vectors on pathogen infected source plants (acquisition access period, AAP) to produce access period response curves (i.e., curves showing the probability of transmission as a function of AAP) (see definitions in Fig 1A). In the second experiment, the experimenter varies the duration of access given to pathogen-bearing vectors on pathogen free source plants (inoculation access period, IAP) to produce inoculation response curves. Varying these durations in this way can also produce plausible ranges for epidemiological parameters such as pathogen retention period. For example, Maruthi *et al*. [1] used access period experiments to conclude that CBSV is effectively acquired within 1 hour (the basis for this conclusion was that no significant differences were found in transmission success for acquisition periods beyond 1 hour) [1]. In addition, Maruthi *et al*. concluded that CBSV has a retention period of less than 48 hours.

A central goal in plant disease epidemiology is the prediction of epidemic risk, and, beyond this, of the prospects for control. The goals are achieved through analyses at the landscape scale, for which estimates of epidemiological parameters are essential, for emerging viral threats such as CBSV. In this paper, we introduce a method for the estimation of epidemiological parameters from access period data. The method consists of first deriving equations for vector infection dynamics as they occur in access period experiments. Solving the equations leads to algebraic expressions for AAP and IAP curves, in which the probability of successful transmission is related to durations of exposure of vectors to a target plant. The algebraic expressions are then matched to empirical curves to estimate the required parameters. We use the method to generate epidemiological parameter estimates for CBSV from a recent access period experiment [1]. Using stochastic simulation, we conduct an initial assessment of the parameter estimates, providing predictions that can be tested empirically.

## Materials and methods

We describe here the typical experimental design of acquisition and inoculation assays, from which we extract a computational and a mathematical model for vector dynamics and vector transmission as well as a corresponding event-based, stochastic, simulation model. Initially we demonstrate the accuracy of our methods using simulated datasets. We finish by applying the methods to a recent CBSV dataset and provide an initial validation.

### Overview of experimental setup (acquisition and inoculation assays)

The virus acquisition assay procedure [9] involves confining a cohort of virus-free insects to an infected plant. After a fixed period of access to the infected plant (acquisition access period) the insects are removed and assessed for infection using a direct or indirect diagnostic test. In the direct test, the proportion of infected vectors is calculated using a molecular diagnostic test such as RT-PCR. In the indirect test, the proportion of infected vectors is calculated by transferring the cohort of insects to a healthy plant for a fixed ‘test’ IAP. The process is performed for a fixed acquisition access period with *n* repetitions, for each of a range of acquisition access periods. In the case of the indirect test, acquisition success corresponds to the proportion of plants from the ‘test’ IAPs that develop symptoms, or, are ELISA-positive (usually after a fixed number of days to allow systemic infection).

The virus inoculation assay procedure involves confining a cohort of potentially virus-bearing insects to a healthy plant for virus inoculation. After a fixed period of access to the healthy plant (inoculation access period), the insects are removed, and the plant is assessed for infection. Inoculation success is evaluated as the proportion of plants showing symptoms [9], or the proportion that are ELISA-positive. Note that for IAP experiments, since the first step involves virus-bearing insects, it is always necessary to place healthy insects on an infected plant for a fixed ‘test’ AAP in advance of the IAP experiment itself. The process is performed for a fixed inoculation access period with *n* repetitions, for each of a range of inoculation access periods.

### Mathematical modelling (vector dynamics, transmission dynamics)

#### AAP model

Following our overview of the acquisition access assay, the AAP model assumes that a cohort of pathogen-free insects, with size denoted by *X*, are given access to a pathogen infected host plant (Fig 1B). The pathogen-free insects acquire the virus at rate *α* (per hour) becoming pathogen-bearing, with size denoted by *Y*. Pathogen-bearing insects lose the pathogen at rate *ν* (per hour) becoming pathogen-free. We additionally allow for a rate of death of insects (per hour), denoted *f*. Combining these simple processes, the probability, *P*_*X*,*Y*_(*t* + *δt*), that there are exactly *X* pathogen-free and *Y* pathogen-bearing insects at time *t* plus small time interval *δt* is:

$$\begin{array}{cc} & \text{Joint}\;\text{probability}\;\text{dynamics}\;\text{of}\;\text{pathogen-free}\;\text{and}\;\text{pathogen-bearing}\;\text{vectors:}\\ & P_{X,Y}\left(t+\delta t\right)=P_{X,Y}\left(t\right)+\overset{\text{Death}}{\overset{︷}{f(\left(X+1\right)P_{X+1,Y}\left(t\right)-XP_{X,Y}\left(t\right)+\left(Y+1\right)P_{X,Y+1}\left(t\right)-YP_{X,Y}\left(t\right))}}\delta t\\ & +\underset{\text{Virus}\;\text{loss}}{\underset{︸}{\nu (\left(Y+1\right)P_{X-1,Y+1}\left(t\right)-YP_{X,Y}\left(t\right))}}\delta t+\underset{\text{Acquisition}}{\underset{︸}{\alpha (\left(X+1\right)P_{X+1,Y-1}\left(t\right)-XP_{X,Y}\left(t\right))\delta t}}. \end{array}$$ (1)

#### IAP model

The IAP model assumes that the population of pathogen-bearing insects from the AAP assay are given access to a healthy host plant (Fig 1C). The number of pathogen-bearing insects are here denoted *y* to distinguish them from the equivalent AAP class (*Y*). All parameters are as for the AAP model, but, in addition, the pathogen-bearing insects inoculate the pathogen into the healthy plant at rate *β* (per hour). Combining these processes the joint probability, *Q*_*M*,*N*_(*t* + *δt*), of exactly *N* pathogen-bearing insects and *M* plant inoculation events at time *t* + *δt* is:

$$\begin{array}{cc} & \text{Joint}\;\text{dynamics}\;\text{of}\;\text{infected}\;\text{plants}\;\text{and}\;\text{pathogen-bearing}\;\text{vectors:}\\ & Q_{M,N}\left(t+\delta t\right)=Q_{M,N}\left(t\right)+\overset{\text{Virus}\;\text{loss}\;\text{or}\;\text{death}}{\overset{︷}{\left(\nu +f\right)(\left(N+1\right)Q_{M,N+1}\left(t\right)-NQ_{M,N}\left(t\right))}}\delta t\\ & +\underset{\text{Inoculation}}{\underset{︸}{\beta (NQ_{M-1,N}\left(t\right)-NQ_{M,N}\left(t\right))}}\delta t, \end{array}$$ (2)

Note that while Eqs 1 and 2 represent mathematical models that are analysed in subsequent sections to gain insight into the underlying dynamics, we also recast the models as event-based processes (using a Gillespie algorithm [10]) in order to simulate epidemics with known parameters from which to test the methods for estimating parameters. The models are subsequently used to estimate parameters from empirical experiments for CBSV and whitefly vectors.

#### Acquisition and inoculation response curves

Equations for both acquisition and inoculation response curves are found by first solving the stochastic process in Eq 1. This involves the application of a number of steps (utilizing probability generating functions and partial differential equations: see S1 Appendix) and shows that the number of pathogen-bearing insects after *t* hours of acquisition access, denoted *y*_0_(*t*), is binomially distributed (with *n* = *X*_0_ and *p*(*t*) = *αe*^−*ft*^(1 − *e*^−(*α*+*ν*)*t*^)/(*α* + *ν*)), see Eq S1.12, S1 Appendix. When acquisition success is determined by molecular testing of individual insects, the acquisition response curve, denoted *P*_*AAP*_, is then simply $\frac{y_{0}\left(t\right)}{X_{0}}=\frac{np(t)}{X_{0}}=p\left(t\right)$, i.e.,

$$\begin{array}{cc} & \text{Acquisition}\;\text{success,}\;\text{with}\;\text{testing:}\;\;\;\;\;\;P_{AAP}\left(t\right)=\frac{\alpha e^{-ft}}{\alpha +\nu }\left(1-e^{-(\alpha +\nu )t}\right). \end{array}$$ (3)

If, instead, molecular diagnostics have not been used, the insects (including *y*_0_ pathogen-bearing insects) are transferred to a healthy plant for a fixed period of inoculation access. The ensuing proportion of plants that become infected is then used as a proxy for acquisition success (cf. ‘indirect assay’, subsection “Overview of experimental setup”). In this case, *y*_0_ (cf. Eq 3) is, on the one hand, the initial condition for the IAP process, and on the other hand, it is a function of the duration of the AAP process. For clarity, henceforth we use the general notation *t*_*A*_ and *t*_*I*_ to represent distinct time variables for acquisition access duration and inoculation access duration respectively. Solving the stochastic process in Eq 2, for *y*_0_(*t*_*A*_) initially pathogen-bearing insects, we find that the probability of plant infection is given by,

$$\begin{array}{c} F\left(t_{I},y_{0}\left(t_{A}\right)\right)=1-(\frac{\nu +f+\beta e^{-\left(\beta +\nu +f\right)t_{I}}}{\beta +\nu +f}{)}^{y_{0}\left(t_{A}\right)}, \end{array}$$ (4)

(S2 Appendix). As we saw (cf. Eq 3), *y*_0_(*t*_*A*_) is itself binomially distributed so that the acquisition response curve, denoted *P*_*AAP*_(*t*_*A*_, *τ*), combines the probability of plant infection, *F*(*t*_*I*_, *y*_0_(*t*_*A*_)) with the binomially distributed initial condition *y*_0_(*t*_*A*_), i.e.,

$$\begin{array}{c} \text{Acquisition}\;\text{success,}\;\text{no}\;\text{testing:}\;\;\;\;P_{AAP}\left(t_{A},\tau \right)=\sum_{k=0}^{X_{0}}\left(\begin{array}{c} X_{0}\\ k \end{array}\right)p{\left(t_{A}\right)}^{k}{\left(1-p\left(t_{A}\right)\right)}^{X_{0}-k}F\left(\tau ,k\right). \end{array}$$ (5)

Thus, the acquisition response curve is described by a single equation that represents a variable acquisition period (duration *t*_*A*_) followed by a fixed inoculation period (duration *t*_*I*_ = *τ*). Analogously, and irrespective of the use of molecular diagnostics, the inoculation response curve, denoted *P*_*IAP*_(*τ*, *t*_*I*_), is described by a single equation representing fixed acquisition period (duration *t*_*A*_ = *τ*) followed by a variable inoculation period (duration *t*_*I*_),

$$\begin{array}{c} \text{Inoculation}\;\text{success:}\;\;\;\;\;\;P_{IAP}\left(\tau ,t_{I}\right)=\sum_{k=0}^{X_{0}}\left(\begin{array}{c} X_{0}\\ k \end{array}\right)p{\left(\tau \right)}^{k}{\left(1-p\left(\tau \right)\right)}^{X_{0}-k}F\left(t_{I},k\right). \end{array}$$ (6)

Details of the analysis used are presented in S3 Appendix. In summary, acquisition and inoculation response curves differ only in that the acquisition period is variable in the latter but constant in the former (*Y*(*t*_*A*_ = *τ*) vs *Y*(*t*_*A*_) respectively) while inoculation period is constant in the latter (*t*_*I*_ = *τ*) but variable in the former (*t*_*I*_). The exception to this pattern occurs when molecular diagnostics have been used, in which case Eq 3 holds instead of Eq 5.

### Combining derived and empirical curves to estimate pathogen parameters

We wish to match the solutions, *P*_*AAP*_(*t*_*A*_, *τ*) and *P*_*IAP*_(*τ*, *t*_*I*_) with corresponding empirical curves. The task is to derive simple formulae for gradients of the curves which can then be easily compared with empirical gradients. This is achieved through differentiation of the acquisition and inoculation response curves and subsequent evaluation at *t*_*A*_ = 0 and *t*_*I*_ = 0, respectively, as set out in S4 Appendix. The resulting formulae for initial slope, initial acceleration and IAP asymptote (by taking the limit as *t*_*I*_ tends to infinity) are listed in Table 1.

**Table 1:** Formulae for gradients of access period response curves. All formulae are derived from equations for acquisition and inoculation response curves in S4 Appendix.

	Gradient #1, initial slope	Gradient #2 (see symbols/caption)
AAP*	*αX*_0_	−(2*f* + *ν* + *α*)
*AAP*^†^	$\alpha X_{0}(1-\frac{\nu +f+\beta e^{-(\nu +f+\beta )\tau }}{\nu +f+\beta })$	−(2*f* + *ν* + *α*)
*IAP*^∼^	*βX*_0_ *p*(*τ*)	$1-(1-\frac{\beta p(\tau )}{\nu +f+\beta }{)}^{X_{0}}$

Table notes AAP* refers to acquisition response curve with molecular diagnostics; *AAP*^†^ refers to acquisition response curve without molecular diagnostics; *IAP*^∼^ refers to inoculation response curve. Note that the inoculation response curve does not involve molecular diagnostics. In the case of: AAP*, Gradient #2 is the ratio of initial acceleration to initial slope; *AAP*^†^, Gradient #2 is the ratio of initial acceleration to initial slope plus scaled initial slope (scaled by (*X*_0_ − 1)/*X*_0_); *IAP*^∼^, Gradient #2 is the asymptote.

#### Examples of simulated dataset and CBSV dataset

In order to demonstrate our methods with known data, we ran one thousand stochastic, event-based simulations with pre-defined epidemiological parameter values, where each simulation consisted of 15 plant/vector cohort repetitions. We calculated AAP and IAP response curves from the means of these simulations over these sets and computed values for initial slope and initial acceleration from the overall mean response curves. This resulted in gradient values that were equated to the formulae in Table 1 and solved for the virus parameters. The entries in Table 1 represent a system of four simultaneous equations in four unknowns that is straightforward to solve. We followed the specific solution order set out in SI section 5 to produce the parameter estimates in Table 2. The procedure that was tested using the simulated dataset was next applied to the access period data of Maruthi *et al*. [1] for CBSV. Note that Maruthi *et al*. did not use molecular diagnostics to confirm insect infection. Since the access period data of Maruthi *et al*. are somewhat noisy (relating to the number of experimental repetitions used), we fit the general form for acquisition success (i.e., transmission success as a function of acquisition period, Eq S3.6, S3 Appendix) to the acquisition access period data, and the general form for inoculation success (i.e., transmission success as a function of inoculation period, Eq S3.5, S3 Appendix) to the inoculation access period data prior to extracting empirical values for initial slope, initial acceleration and asymptote (i.e., the empirical gradients). Using the same proceedure that we followed for the simulated dataset, these empirical gradients were then matched to the theoretical gradients in Table 1 to produce the parameter estimates in Table 3 (i.e., we solved the resulting simultaneous equations using the particular order of steps set out in S5 Appendix).

**Table 2:** Estimates of epidemiological parameters from a simulated access period experiment.

Simulated dataset	Epidemiological parameter	True value	Estimated value
**no diagnostics**	Rate of virus loss, *ν*	0.2*h*^−1^	0.197*h*^−1^
	Rate of virus acquisition, *α*	0.05*h*^−1^	0.055*h*^−1^
	Rate of virus inoculation, *β*	0.033*h*^−1^	0.029*h*^−1^
**with diagnostics**	Rate of virus loss, *ν*	0.2*h*^−1^	0.199*h*^−1^
	Rate of virus acquisition, *α*	0.05*h*^−1^	0.049*h*^−1^
	Rate of virus inoculation, *β*	0.033*h*^−1^	0.033*h*^−1^

Table notes Simulations were initiated under the assumption that in *no diagnostics*: no molecular diagnostics were available, and in *with diagnostics*: molecular diagnostics were used. Epidemiological parameters were estimated by matching the means of 1000 simulated acquisition and inoculation response curves, with each simulation consisting of 15 replicates of an individual plant exposed to 50 insects, in *no diagnostics*: to the gradients of *AAP*^†^ and *IAP*^∼^, and in *with diagnostics*: to the gradients of AAP* and *IAP*^∼^ (Table 1, gradient #1-#2). Note that in both scenarios the parameter estimate for the additional death rate, *f*, was close to the true value (true value 0.02*h*^−1^; estimate in A: 0.018*h*^−1^; estimate in B: 0.02*h*^−1^).

**Table 3:** Epidemiological parameter estimates for CBSV: An application of the method of matching gradients to a CBSV access period dataset.

CBSV dataset [1]	Epidemiological parameter	Estimated value
	Retention period, $\frac{1}{\nu }$	95*mins*
	Acquisition period, $\frac{1}{\alpha }$	33*mins*
	Inoculation period, $\frac{1}{\beta }$	48*h*

Table notes Epidemiological parameters were estimated by matching empirical acquisition and inoculation response curves to the *AAP*^†^ and *IAP*^∼^ gradients (#1-#2), respectively.

#### Validation of parameter estimates for CBSV

Maruthi *et al*. describe a further two experiments involving access period arrays beyond the basic access period experiment: ‘transmission’ and ‘persistence’ experiments [1]. The first was designed to assess ‘transmission’ as the cohort size of insects is increased for fixed acquisition and inoculation access periods. In the second experiment, ‘persistence’ of the virus in an insect vector is measured by exposing the insects to a sequence of three plants; the original infected plant, a dummy healthy plant and finally the success of transmission is tested on a third, healthy plant. By allowing the insects to feed on the intermediate healthy plant (for potentially different exposure times) the persistence of the virus in the vector could be assessed.

As an initial test of the CBSV parameter estimates, we ran dynamic simulations of both the ‘transmission’ and ‘persistence’ experiments. This involved assigning our CBSV parameter estimates to the epidemiological parameters and reproducing the structure of these experiments as event-based stochastic simulations.

## Results

In this section we begin by examining the response curves that result from both computer simulation and mathematical analysis. In addition, the results of applying the formulae, which were derived from the mathematical analysis, to the data from the computer simulations are presented. We then present epidemiological parameter estimates that follow from the application of the method to CBSV data. Finally, making use once again of computer simulations, the results of preliminary testing of the CBSV estimates are presented.

**Fig 2:**
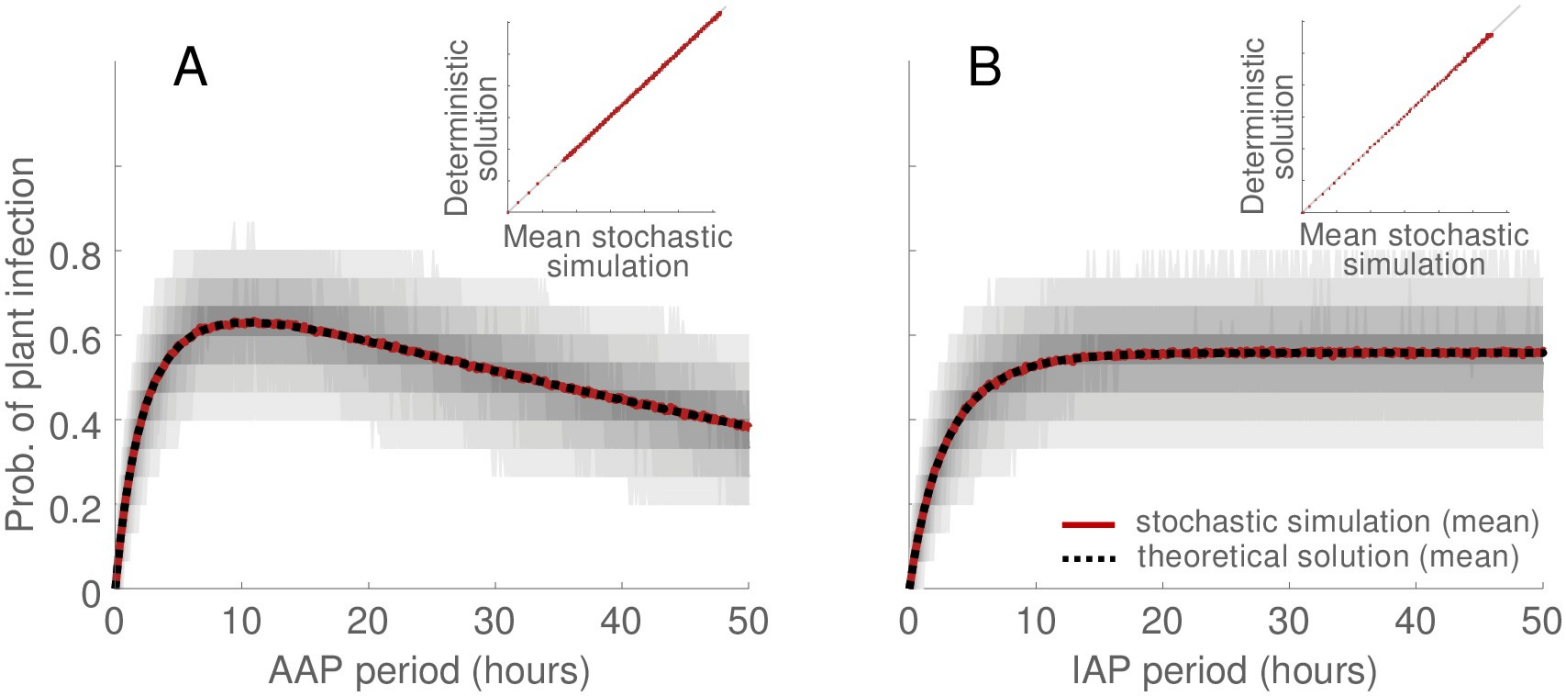
Stochastic modelling of access period assays without molecular diagnostics. In A: acquisition response curve, in B: inoculation response curve. In A-B gray curves indicate the 2.5th-97.5th percentile range of simulations (i.e., demarcating 95% of simulations with darker shades of gray for percentile bands near the median). Solid ruby curves indicate the mean of simulations. Dotted black curves show the mathematical solution (i.e., Eq 5 in A, and Eq 6 in B). Figure inset shows the correspondence between computer simulations and mathematical solutions. Computer simulation involved reproducing the access period experiments using event-based stochastic simulation, with the epidemiological parameters assigned the values listed in the ‘True value’ column of Table 2. In both A and B 1000 simulations were initiated with cohorts of 50 virus-free adult vectors placed on virus-infected plants, with 15 plant/insect cohort replicates per simulation. The fixed access period duration was 24 *hours* (i.e., the inoculation access period in *A*, and the acquisition access period in *B*, was 24 *hours*).

**Fig 3:**
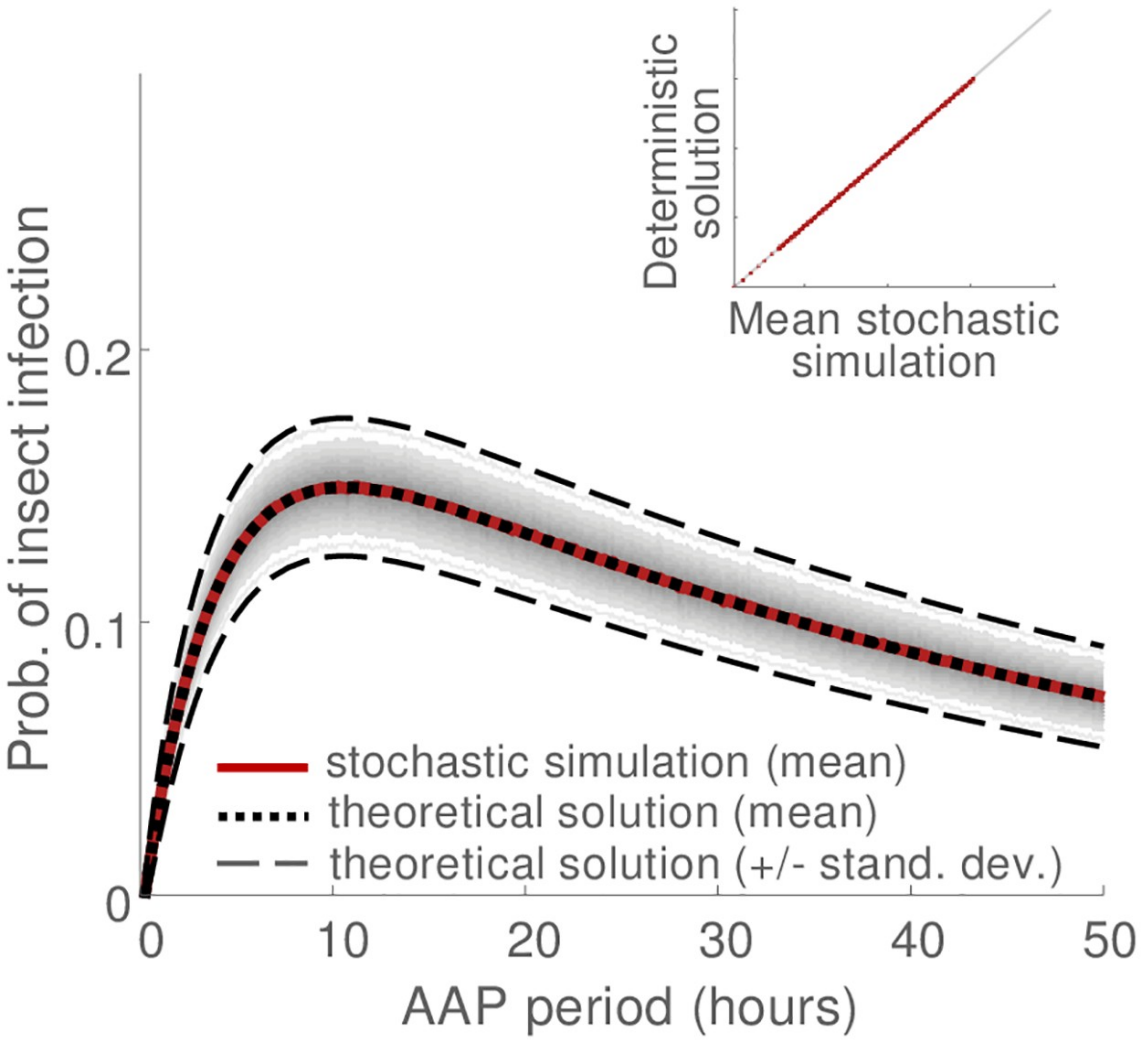
Stochastic modelling of access period assays with molecular diagnostics, acquisition response curve. Note that the use of molecular diagnostics to assess insect infection affects only the acquisition access component of access period assays, see Fig 2B for corresponding inoculation response curve. Gray curves indicate the 2.5th-97.5th percentile range of simulations (i.e., demarcating 95% of simulations with darker shades of gray for percentile bands near the median). Solid ruby curve indicates the mean of simulations. The black dotted curve shows the mathematical solution (i.e., Eq 3). In addition, black dotted curves indicate the mathematical solution +/- the derived standard deviation (see S1 Appendix for derivation of variance). Figure inset shows the correspondence between computer simulations and the mathematical solution. For details of the simulation procedure see caption of Fig 2.

The mathematical equations (Eqs 3, 5 and 6) for the access period response curves closely match the overall mean of the stochastic simulations (Figs 2 and 3, dotted black vs solid ruby curves). This is the case when molecular diagnostics were not used (Fig 2) and when they were used (Fig 3). The correspondence between the theoretical and simulation results demonstrates the accuracy of the mathematical solutions. The mathematical solutions for the access period response curves were distilled in to formulae for curve gradients, forming a set of simultaneous equations for the unknown epidemiological parameters when equated to the empirical gradients. We found that for acquisition response curves the gradients in which the epidemiological parameters were expressed most simply were initial slope and the ratio of initial acceleration to initial slope (Table 1), while for inoculation response curves they were expressed most simply in terms of the initial slope and the asymptote (Table 1). Solving the system of simultaneous equations results in the epidemiological parameter estimates. Comparing these estimates with their true values for the simulated data, we find that the estimates for acquisition period, inoculation period and retention period are very accurate (Table 2). This was the case both when we reproduced the situation in which no molecular diagnostics were available (Table 2, no diagnostics), and when we reproduced the situation in which molecular diagnostics were used (Table 2, with diagnostics). The estimates were marginally more precise when molecular diagnostics were used than when they were not used (Table 2, with diagnostics).

We then applied the procedure to the data of Maruthi *et al*. [1] in which a CBSV isolate from southern, coastal Tanzania was tested in relation to cassava host plants using insects of a *B. tabaci* strain known to be of subgroup SSA1-SG1 [1]. When the procedure, i.e., the method of matching gradients, was applied to the data of Maruthi *et al*. [1], the mean retention period was found to be approximately 1.6*h*. In addition, we found that the mean acquisition period was approximately 0.55*h* and the mean inoculation period was approximately 48*h* (units of expected duration; Table 3). At first sight a 48*h* inoculation period appears surprisingly long, however, in reality this is an expected value that comprises not only the length of time it takes for inoculation processes to occur, but also the likelihood that inoculation events actually lead to plant infection. In addition, the death rate, *f*, which was found to be very close to zero (≈ −0.003), was set to zero here and in subsequent dynamic simulations.

**Fig 4:**
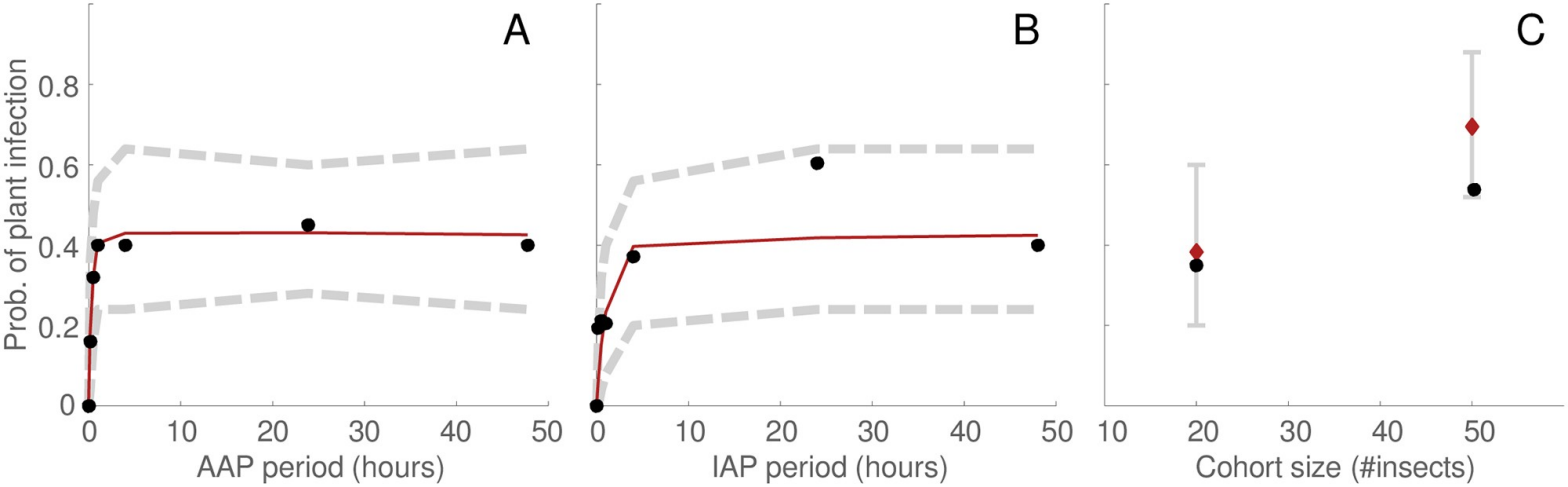
An empirical test of the epidemiological parameter estimates for CBSV: Dynamic simulation vs experimental data for variable acquisition period (A), for variable inoculation period (B), and for variable initial insect density (C). In A-C ruby curves/diamonds indicate the mean of simulations, gray curves/intervals indicate the 2.5th and 97.5th percentiles of simulations (i.e., demarcate 95% of simulations). Black circles indicate data-points from Maruthi *et al*. [1]. In A-C, dynamic simulations were implemented using the epidemiological parameter estimates for CBSV in Table 3. For details of the simulation procedure see caption of Fig 2.

**Fig 5:**
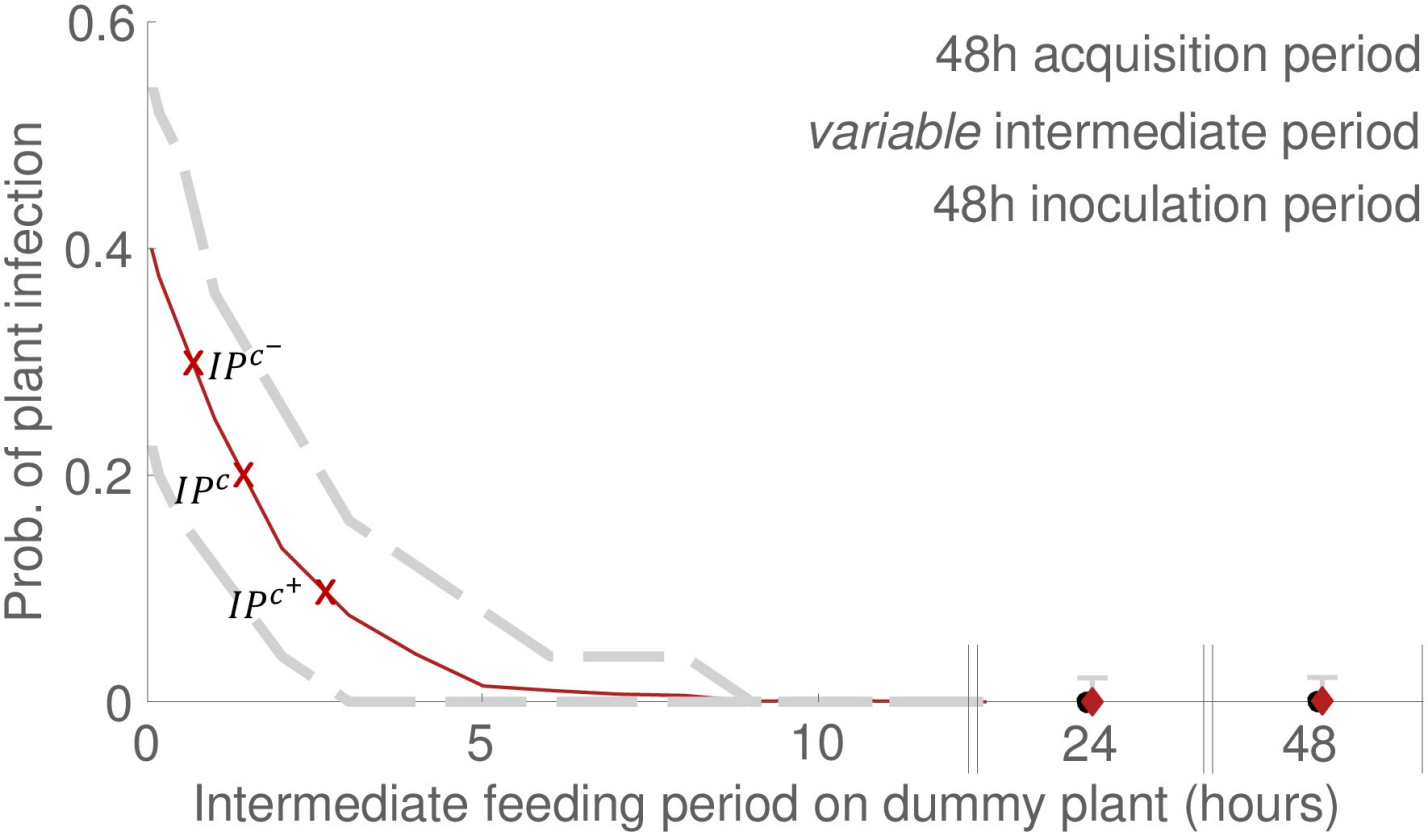
An empirical test of epidemiological parameter estimates for CBSV: Dynamic simulations vs. experimental data for variable intermediate feeding periods. An additional experiment is described in Maruthi *et al*. in which insects were given access to an intermediate feeding period on a dummy plant [1] (see methods section). Black circles indicate datapoints from [1]. Ruby curves/diamonds indicate the mean of simulations. Gray dashed curves/gray intervals indicate the 2.5th and 97.5th percentiles of simulations. Red crosses mark the intermediate period at which transmission success was 50% of that of no intermediate period (*IP*^*c*^: 0.197 prob. of plant infection after 1.45*h* intermediate period), 25% of that of no intermediate period ($IP^{c^{+}}$: 0.099 prob. of plant infection after 2.58*h* intermediate period), and 75% of that of no intermediate period ($IP^{c^{-}}$: 0.296 prob. of plant infection after 0.6*h* intermediate period)(c.f. prob. of plant infection was 0.394 when the intermediate period was 0*h*). For details of the simulation procedure see caption of Fig 2. Note that Maruthi *et al*. considered intermediate periods of 24 and 48 hours (appended portions of x-axis), see S6 Appendix. At these points the gray vertical simulated intervals consist only of the zero value for the probability of plant infection.

Using CBSV parameter estimates (i.e., *α*, *ν*, *β* and *f*) from the access period experiment of Maruthi *et al*. [1] as the basis of dynamic stochastic simulations, we generated a 2.5^*th*^-97.5^*th*^ percentile range (i.e., demarcating the 95% credible region of response curves) as a function of acquisition period and inoculation period (Fig 4A and 4B, gray dashed curves). We found that the datapoints [1] fell comfortably within the predicted range (Fig 4A and 4B, black circles). In addition to the basic access period experiment, we also implemented dynamic simulation of two additional experiments. In the first of these, a ‘transmission’ experiment, the number of insect vectors used in the basic access period setup is varied. The dynamic simulations generated a 2.5^*th*^-97.5^*th*^ percentile range for the two cohort sizes studied (Fig 4C, gray intervals) and we found that the datapoints [1] fell within the range (Fig 4C, black circles). In the second experiment, designed to measure ‘persistence’ of the pathogen in the vector, the duration of an intermediate access period on a dummy plant was varied in our simulations. The dynamic simulations generated a 2.5^*th*^-97.5^*th*^ percentile range for the two intermediate periods (Fig 5, gray dashed curves for intermediate period < 15*h*; gray interval on the appended x-axis i.e., intermediate period > 15*h*), and we found that the datapoints [1] exactly matched the predicted ranges (Fig 5, black circles on appended x-axis; note that the simulation percentile ranges included only the zero value for transmission success).

It may be the case that conventional expectations of CBSV retention period governed the choice of the intermediate feeding duration studied in [1]. Taken together, the zero datapoints in the Maruthi *et al*. experiment [1], i.e., zero plants infected after 24*h* and 48*h* feeding on an intermediate dummy plant, and the retention period estimate of 1.6*h* that is presented here, show that the chosen intermediate period was long relative to the duration of persistence. In order to propose a more plausible range of intermediate periods, and to provide a means of empirically testing our findings, we produced credible 95% intervals for all values of the intermediate period (Fig 5). We labeled several key points on the mean response curve, i.e., critical intermediate durations at which the persistence response was 25%, 50% and 75% of the case when no intermediate period was used (see caption of Fig 5 for exact values).

## Discussion

Typically, small-scale laboratory experiments, such as experiments in vector access to host plants, are employed to investigate epidemiological parameters such as retention period (of the pathogen in the vector). In this paper, we have derived explicit solutions for the acquisition and inoculation response curves that these experiments produce, which we reduced to a set of formulae for curve gradients. The formulae, when compared with empirical gradient values, are used to estimate epidemiological parameters. The method proved very accurate when tested on simulated datasets (for which the true parameter values were known). To date, there remains much to be learned concerning the detailed epidemiology of *Cassava brown streak virus* (CBSV) that is spreading on an east-west axis across sub-Saharan Africa, and is currently threatening regional food security. We applied our methods to a recent CBSV access period dataset [1] and found that CBSV is retained for a far shorter duration in its insect vector (*Bemisia tabaci* whitefly) than had previously been assumed. We also found from dynamic simulations of the access period experiments that our estimate for retention period is consistent with the additional ‘transmission’ and ‘persistence’ experiments reported in Maruthi *et al*. [1].

### The implications of the epidemiological parameter estimates for CBSV

For most of its known history, CBSV was confined to coastal East Africa and the shores of Lake Malawi but more recently the virus has been undergoing a dramatic increase in geographic range [5]. In general, infectious disease epidemics depend on *R*_0_, the pathogen’s basic reproduction number (i.e. the average number of secondary cases produced by a primary case in a susceptible population). In particular, a condition of epidemic spread is that *R*_0_ > 1, while epidemic fade-out occurs if *R*_0_ < 1. In epidemiological terms it is reasonable to suppose that some component of the *R*_0_ for CBSV in sub-Saharan Africa has changed in recent decades, and, as a consequence, the sub-threshold dynamic (i.e., where *R*_0_ < 1 outside of the confined coastal East Africa and the shores of Lake Malawi regions), has transitioned to a dynamic of super-threshold spread (i.e., *R*_0_ > 1). It is clear that the retention, acquisition and inoculation periods, together with the density of whitefly are all critical components of *R*_0_. Quantifying these epidemiological parameters is therefore an important step in understanding CBSV range expansion.

Several observations concerning CBSV range expansion can be made using the parameter estimates presented in this work. Firstly, the short retention period (1.6 *hours*) is consistent with sub-threshold (i.e. non-epidemic) epidemiology of CBSV since it is associated with a brief whitefly infectious period. The inoculation period is the inverse of the *rate of successful inoculation* (per infected whitefly), which is itself composed of the rate that the virus is introduced into the plant during feeding scaled by the probability that inoculation events lead to plant infection. The long inoculation period (relative to retention period) reported here (48 *hours* cf. 1.6 *hours*) is another factor in sub-threshold *R*_0_. It is likely to reflect a low frequency with which inoculation events are successful (i.e., result in plant infection). All of the parameter estimates derived above are consistent with the conclusions of Maruthi *et al*. [1], who found that retention period is < 48 *hours*, acquisition period is < 1 *hour* and CBSV is inoculated in a manner that increases up to 24 *hours*. Our findings extend the results of Maruthi *et al*. [1] by providing parameter estimates (rather than plausible ranges) that can be used for epidemiological applications such as landscape simulation. As with Maruthi *et al*. [1]’s experiment, and in spite of the constraints of a short retention period and a long inoculation period, substantial plant infection does occur in our model of access period assays. In part, this is facilitated by rapid acquisition of the virus when whitefly feed on infected plants (with an acquisition period of 0.55 *hours*), and in part this is due to the relatively high density of whitefly used in the experiments.

In this paper we have introduced a method of matching gradients in order to estimate epidemiological parameters from conventional access period experiments. The method can be used for a wide range of plant viruses using data available in published experiments. Viruses like CBSV do not have a latent period in their vector and are restricted to the phloem of the host plant (i.e., ‘semi-persistent’ viruses [11]), and hence are acquired and inoculated during feeding from phloem (as depicted in Fig 1, Eqs 1 and 2). The method can also be applied to phloem-restricted viruses that are associated with periods of latency in the insect vector, by extending Eqs 1 and 2 to account for latency (such viruses include ‘persistently transmitted’ viruses like cassava mosaic virus [11, 12]). Viruses that are acquired and inoculated from the host plant’s epidermal cells by aphid vectors, however, require a different set of equations for plant infection that take account of the central role of aphid probing in acquisition and inoculation (i.e., non-persistently transmitted viruses; see Donnelly *et al*. [13] for a model that could be adapted for this purpose). In order to provide a means of testing the parameter estimates it may be useful to generate secondary predictions using dynamic simulation. In this paper we used the estimates to predict the relationship between the probability of plant infection and the duration of feeding on a dummy plant intermediate to a 48 *hour* acquisition period and a 48 *hour* inoculation period. Accordingly, by identifying the intermediate period at which plant infection was 25%, 50%, and 75% of that of no intermediate period (Fig 5), we provided a predicted range that can be empirically tested.

How do our findings relate to epidemics in the field? Our results shed light on a number of critical factors that may be responsible for the transition of CBSV from sub- to super-threshold *R*_0_ in sub-Saharan Africa. These factors include *a*): pathogenic adaptation to the cassava host such that the frequency of plant infection following inoculation is increased, *b*): the invasion of novel strains of the *B. tabaci* whitefly having greater affinity for the cassava host, such that insect vectors spend longer periods of time feeding from cassava phloem. CBSV like other RNA viruses, evolves quickly [14], so that CBSV may have evolved to overcome host defences [15]. A number of *B. tabaci* strains collected from cassava have been shown to vary in host preference and range of diet [16]. This, in turn, suggests that geographic variation in *B. tabaci* strains [17] is likely to be associated with variation in *B. tabaci* feeding behavior [15]. In addition to these possible factors, the increasing abundance of whitefly across Eastern sub-Saharan Africa since the 1990s [5, 18–21] is certain to have played a key role. Notably the confirmation of re-emergence of CBSD in central Uganda coincided with generally high *B. tabaci* populations in the area [2]. Our results also point to the importance of infected cuttings in determining the rate of spread of the epidemic front. The shorter the retention period, the shorter is the distance over which the virus can be effectively carried by insects in airflows to infect the host plant. This then suggests that long distance transmission of CBSV is likely to be dominated by human-mediated movement of infected cuttings rather than by insect movement. However, it should be borne in mind that even for the very briefly retained, non-persistently transmitted potyviruses there is evidence in the literature that some long-distance air-borne dispersal does occur [22].

The critical factors that may be responsible for the transition of CBSV from sub- to super-threshold *R*_0_ in sub-Saharan Africa will be explicitly investigated in future work using an epidemiological model for CBSV transmission within fields of cassava plants. By expressing *R*_0_ in terms of the epidemiological parameter estimates presented here, the role of each factor in CBSV’s transition from sub- to super-threshold spread (i.e., from *R*_0_ < 1 to *R*_0_ > 1) can be assessed. In particular, the critical abundance of the whitefly vector that is needed for epidemics to occur can be quantified. Ultimately, such studies will help identify those aspects of CBSV epidemiology that are most critical to epidemic spread, and will lead to management recommendations for containing the geographic range of CBSV.

## Supporting information

S1 Appendix Stochastic modelling of experiments in vector acquisition access to host plants. (PDF)

S2 Appendix Stochastic modelling of experiments in vector inoculation access to host plants. (PDF)

S3 Appendix Equations for acquisition and inoculation response curves; remarks on the use of molecular diagnostics. (PDF)

S4 Appendix Method of matching gradients, deriving formulae for the gradients of response curves. (PDF)

S5 Appendix Steps taken to estimate epidemiological parameters. (PDF)

S6 Appendix Data appearing in the access period experiment of Maruthi *et al*. (2016). (PDF)
