# Supplementary material for: Estimating epidemiological parameters from experiments in vector access to host plants, the method of matching gradients
Source: PLoS Comput Biol. 2020 Mar 16;16(3):e1007724. doi: 10.1371/journal.pcbi.1007724 (PMC7098647; doi:10.1371/journal.pcbi.1007724)
Supplement: S1 Appendix — (PDF) [file pcbi.1007724.s001.pdf]

In the following set of appendices, we first derive explicit solutions from the vector equations in the main text for acquisition access arrays (S1 Appendix) and then for inoculation access arrays (S2 Appendix). This essentially results in a pair of equations for access period response curves (S3 Appendix). We reduce these equations further by deriving formulae for two gradients from each curve: initial acceleration and initial slope of acceleration as well as asymptote in the case of the inoculation response curve (S4 Appendix). The utility of these formulae is that they can be directly compared with empirical gradient values from published curves to infer virus life-histories. Comparison of empirical and theoretical gradients result in four simultaneous equations with four epidemiological unknown variables (i.e., rate parameters). Hence, it is straightforward to estimate these parameters from the simultaneous equations. In S5 Appendix we set out the particular steps that we took. In S6 Appendix we summarize the dataset from the CBSV laboratory experiment of Maruthi *et al.*(1).

S1 Appendix, Stochastic modelling of experiments in vector acquisition access to host plants

The equations governing the feeding dynamics of insect vectors in an acquisition access period (AAP) assay are,

Joint probability dynamics of virus-free and virus-bearing vectors $P_{X,Y}(t+\delta t)$

$$= P_{X,Y}(t) + \Bigg(\overbrace{f(X+1)P_{X+1,Y}(t) - fXP_{X,Y}(t) + f(Y+1)P_{X,Y+1}(t) - fYP_{X,Y}(t)}^{\text{Death}} \quad \text{(S1.1)}$$
$$+ \underbrace{\nu(Y+1)P_{X-1,Y+1}(t) - \nu Y P_{X,Y}(t)}_{\text{Virus loss}} + \underbrace{\alpha(X+1)P_{X+1,Y-1}(t) - \alpha X P_{X,Y}(t)}_{\text{Acquisition loss}} \Bigg)\delta t$$

where X and Y represent the number of virus-free and virus-bearing insect vectors. System S1.1 has the initial condition: $X(0) = X_0, Y(0) = 0$ (i.e., $P_{X_0,0}(0) = 1$). Insects acquire the virus at per capita rate α (per hour), lose the virus at per capita rate ν (per hour) and die at per-capita rate f (per hour), as per main text.

System S1.1 can be rewritten as a partial differential equation (PDE) in which the dependent variable is the probability generating function, denoted $g(z_1, z_2, t)$, of the variables X and Y from

system S1.1, i.e., $g(z_1, z_2, t) = \sum_{X,Y} z_1^X z_2^Y P_{X,Y}(t)$. The strategy is to solve the PDE and hence to recover the distribution of either population variable by manipulating the system's probability generating function. Multiplying both the left hand side and right hand terms of the stochastic process in Eq. 1 by $\sum_{X,Y} z_1^X z_2^Y$ and rearranging, produces the PDE:

$$\frac{\partial g}{\partial t} = \frac{\partial g}{\partial z_1}(f + \alpha z_2 - (f+\alpha)z_1) + \frac{\partial g}{\partial z_2}(f + \nu z_1 - (f+\nu)z_2) \tag{S1.2}$$

The PDE in Eq. S1.2 is linear and can be solved along characteristic curves (curves on which the solution $g(z_1, z_2, t)$ is constant). This involves forming a linear system of ODEs from the PDE. They are given by,

$$\begin{aligned} \frac{dz_1}{dt} &= -(f + \alpha z_2 - (f+\alpha)z_1) \\ \frac{dz_2}{dt} &= -(f + \nu z_1 - (f+\nu)z_2) \end{aligned} \tag{S1.3}$$

This linear ODE system can be solved by first changing the variables so it becomes a homogeneous system and then finding the eigenvalues of the coefficient matrix A. In summary the variables change according to $\mu_1 = -\alpha\nu + f(f+\nu)/((f+\nu)(f+\alpha))$, $\mu_2 = -\alpha\nu + (f(f+\alpha) + \nu f)/((f+\nu)(f+\alpha))$ and $r = t$. The coefficient matrix is then,

$$A = \begin{pmatrix} f+\alpha & -\alpha \\ -\nu & f+\nu \end{pmatrix}$$

which has the eigenvalues: $\lambda_1 = f$, $\lambda_2 = f + \alpha + \nu$. By the principle of superposition the

solutions of the ODE system are,

$$\mu_1(r) = \delta_1 e^{fr} + \delta_2 e^{(f+\alpha+\nu)r} \tag{S1.4}$$

$$\mu_2(r) = \kappa_1 e^{fr} + \kappa_2 e^{(f+\alpha+\nu)r} \tag{S1.5}$$

Then, letting $\mu_1(0) = \mu_1^0$ and $\mu_2(0) = \mu_2^0$,

$$\mu_1(r) = e^{fr}(\frac{\alpha}{\alpha+\nu}(\mu_1^0 - \mu_2^0)(e^{(\alpha+\nu)r} - 1) + \mu_1^0 \tag{S1.6}$$

$$\mu_2(r) = e^{fr}(\frac{\nu}{\alpha+\nu}(\mu_2^0 - \mu_1^0)(e^{(\alpha+\nu)r} - 1) + \mu_2^0 \tag{S1.7}$$

Next we rearrange the solution equations to isolate the constant terms on one side of the equals sign. By the method of characteristics, the PDE solution, and hence the generating function, is some function of the right hand sides, i.e.,

$$\begin{aligned} g(z_1, z_2, t) &= F(z_0^1, z_0^2) \\ &= F(e^{-ft}[z_1 - 1 + \frac{\alpha}{\alpha+\nu}(z_1 - z_2)(e^{(\alpha+\nu)t} - 1)], \\ &\quad e^{-ft}[z_2 - 1 + \frac{\nu}{\alpha+\nu}(z_1 - z_2)(e^{(\alpha+\nu)t} - 1)]) \end{aligned} \tag{S1.8}$$

where we have reversed the change of variables from μ back to z. All that remains is to find the function F and this is achieved by using the generating function's initial condition, i.e. $g(z_1, z_2, 0) = z_1^{X_0}$ so that,

$$F(z_0^1, z_0^2)\Big|_{t=0} = z_1^{X_0} \tag{S1.9}$$

Evaluating the first argument to F at $t = 0$, letting it equal to a dummy variable x, solving x in terms of z_1, and finally replacing z_1 with the resulting expression leads to,

$$g(z_1, z_2, t) = (1 + e^{-ft}(z_1 - 1 + \frac{\alpha}{\alpha + \nu}(z_1 - z_2)(e^{(\alpha+\nu)t} - 1)))^{X_0}. \tag{S1.10}$$

Next we recall that we are interested in Y (i.e., the number of virus-bearing insects), and hence we reduce Eq. S1.10 to a generating function in Y only, i.e.,

$$G(z, t) = g(z_1, 1, t) = (1 + e^{-ft}(z - 1 + \frac{\alpha}{\alpha + \nu}(z - 1)(e^{(\alpha+\nu)t} - 1)))^{X_0} \tag{S1.11}$$

Finally, taking the nth derivative over z of $G(z, t)$ and evaluating at $z = 0$ (which produces the probability that $Y = n$), we see that the underlying distribution for the variable Y after t hours of acquisition access has the binomial form,

$$\begin{aligned}\frac{dG^{(n)}(0,t)}{dt} &= \frac{X_0(X_0-1)...(X_0-n+1)}{n!} p(t)^n (1 - p(t))^{x_0 - n} \\ &= \sum_k \binom{X_0}{k} p(t)^k (1 - p(t))^{X_0 - k} \end{aligned} \tag{S1.12}$$

where $p(t) = \frac{\alpha}{\alpha+\nu}(e^{-ft} - e^{-(f+\alpha+\nu)t})$. The mean and variance of a binomial distribution with parameters n and p, are np and $np(1 - p)$ respectively, and hence mean and variance of the number of virus-bearing insect vectors are,

$$\langle Y \rangle = \frac{\alpha X_0}{\alpha + \nu}(e^{-ft} - e^{-(f+\alpha+\nu)t}) \tag{S1.13}$$

$$Var(Y) = \frac{\alpha}{\alpha + \nu}(e^{-ft} - e^{-(f+\alpha+\nu)t})(1 - \frac{\alpha}{\alpha + \nu}(e^{-ft} - e^{-(f+\alpha+\nu)t})) \tag{S1.14}$$

References

[1] Maruthi MN, Jeremiah SC, Mohammed IU, Legg JP. The role of the whitefly, Bemisia tabaci (Gennadius), and farmer practices in the spread of cassava brown streak ipomoviruses. J Phytopathol. 2016; 31: 1–11.
