# Supplementary material for: Estimating epidemiological parameters from experiments in vector access to host plants, the method of matching gradients
Source: PLoS Comput Biol. 2020 Mar 16;16(3):e1007724. doi: 10.1371/journal.pcbi.1007724 (PMC7098647; doi:10.1371/journal.pcbi.1007724)
Supplement: S2 Appendix — (PDF) [file pcbi.1007724.s002.pdf]

S2 Appendix, Stochastic modelling of experiments in vector inoculation access to host plants

The equation describing the joint probability, $Q(t+\delta t)$, of exactly N pathogen-bearing insects and M plant inoculation events at time $t+\delta t$ in an inoculation access period assay (IAP) is,

Joint dynamics of infected plants and virus-bearing vectors $Q_{M,N}(t+\delta t) = Q_{M,N}(t)$

$$+\left(\overbrace{(\nu+f)(N+1)Q_{M,N+1}(t)-(\nu+f)NQ_{M,N}(t)}^{\text{Virus loss or death}}+\overbrace{\beta N Q_{M-1,N}(t)-\beta N Q_{M,N}(t)}^{\text{Inoculation}}\right)\delta t \tag{S2.1}$$

with initial conditions: $M(0)=0, N(0)=y_0$ (i.e., $Q_{0,y_0}(0)=1$). The system can be rewritten as a partial differential equation (PDE) in which the dependent variable is the probability generating function, denoted $w(s_1,s_2,t)$, of the variables M and N from system S2.1, i.e., $w(s_1,s_2,t) = \sum\limits_{M,N} s_1^M s_2^N Q_{M,N}(t)$. Multiplying both the left hand side and right hand terms of the process in Eq. S2.1 by $\sum\limits_{M,N} s_1^M s_2^N$ and rearranging, produces the PDE,

$$\frac{\partial w}{\partial t} = \frac{\partial w}{\partial s_1}0 + \frac{\partial w}{\partial s_2}((\nu+f)(1-s_2)-\beta s_2(1-s_1)) \tag{S2.2}$$

The PDE given by Eq. S2.2 is linear and can be solved along characteristic curves (curves on which the solution $w(s_1,s_2,t)$ is constant). This involves forming a linear system of ODEs from the PDE. They are given by,

$$\frac{ds_1}{dt} = 0$$
$$\frac{ds_2}{dt} = (\beta s_2(1 - s_1)) - ((\nu + f)(1 - s_2) \quad \text{(S2.3)}$$

Thus s_1 is constant with respect to time, and s_2 is governed by a linear ODE which can be solved by the change of variables so it becomes homogeneous. In summary the variables change according to $s_1' = s_1$ and $s_2' = s_2 - \hat{s}_2$, $b = t$ where $\hat{s}_2 = (\nu + f)/(\beta(1 - s_1) + \nu + f)$ (recalling that s_1 is constant with respect to time). The solution of the ODE is,

$$s_2'(b) = {s_2'}^0 e^{\beta(1-\hat{s}_1)+f+\nu)b} \quad \text{(S2.4)}$$

where we have let $\hat{s}_2'(0) = \hat{s}_2'^0$. Hence,

$$s_2^0 = \hat{s}_2 + e^{-(\beta(1-\hat{s}_1)+f+\nu)b}(s_2 - \hat{s}_2) \quad \text{(S2.5)}$$

and hence,

$$w(s_1, s_2, t) = H(s_0^1, s_0^2)$$
$$= H(s_1, \hat{s}_2 + (s_2 - \hat{s}_2)e^{-(\beta(1-z_1^0+\nu+f)b}) \quad \text{(S2.6)}$$

All that remains is to find the function H and this is achieved by using the generating function's initial condition, i.e. $w(s_1, s_2, 0) = s_2^{y_0}$ so that,

$$H(s_0^1, s_0^2)\Big|_{t=0} = s_2^{y_0} \tag{S2.7}$$

Evaluating the second argument to H at $t = 0$, letting it equal to the dummy variable y, solving y in terms of s_2 on the right hand side of Eq. S2.7, and finally replacing s_2 with the resulting expression, leads to,

$$w(s_1, s_2, t) = (\hat{s}_2 + (s_2 - \hat{s}_2)e^{-(\beta(1-s_1)+\nu+f)t})^{y_0} \tag{S2.8}$$

with $\hat{s}_2 = (\nu + f)/(\beta(1 - s_1) + \nu + f)$. Note that $\hat{s}_2 = 1$ when $s_1 = 1$. Next we recall that we are interested chiefly in N (i.e., the number of plant inoculations), and hence we reduce Eq. S2.7 to a generating function in N only, i.e.,

$$W(s,t) = w(1, s_2, t) = \left(\frac{\nu + f + \beta(1 - s_1)e^{-\beta(1-s_1)+\nu+f)t}}{(\beta(1 - s_1) + \nu + f} \right)^{y_0}. \tag{S2.9}$$

since $w(1, s_2, t) = \sum_{M,N} 1^M s_2^N P_{M,N}(t) = w(s_2, t)$ by the definition of generating functions. Since we are interested in the probability of plant infection, denoted $S(t)$, we can finally convert Eq. S2.9 to a simpler form by calculating the probability that $N \geq 1$. This leads to,

$$S(t) = 1 - W(0,t) = 1 - \left(\frac{\nu + f + \beta e^{-(\beta+\nu+f)t}}{\beta + \nu + f} \right)^{y_0}. \tag{S2.10}$$
