# Supplementary material for: Estimating epidemiological parameters from experiments in vector access to host plants, the method of matching gradients
Source: PLoS Comput Biol. 2020 Mar 16;16(3):e1007724. doi: 10.1371/journal.pcbi.1007724 (PMC7098647; doi:10.1371/journal.pcbi.1007724)
Supplement: S3 Appendix — (PDF) [file pcbi.1007724.s003.pdf]

S3 Appendix, Equations for acquisition and inoculation response curves; remarks on the use of molecular diagnostics

A difficulty that arises in the analysis of access period experiments is that in some cases acquisition efficiency is directly investigated by applying molecular diagnostics to insects after a period of acquisition access. In other cases, however, no molecular diagnostics are available, and an additional step is required: potentially virus-bearing insects are given a fixed period of inoculation access to indirectly investigate acquisition efficiency.

The essential output after t_A hours of acquisition access is a distribution for the number of virus-bearing insect vectors i.e., the probability of k virus-bearing insects is,

$$P_k(t_A) = \binom{X_0}{k} p(t_A)^k (1 - p(t_A))^{X_0 - k} \qquad \text{(S3.1)}$$

where $p(t_A) = \frac{\alpha}{\alpha+\nu}(e^{-ft_A} - e^{-(f+\alpha+\nu)t_A})$, see Eq. S1.12. Where molecular diagnostics have been used, the acquisition response curve is then the fraction of total vectors that are confirmed as virus-bearing, i.e., the mean of the distribution (Eq. S3.1) scaled by the initial number of insects X_0:

Acquisition success, with testing $$P_{AAP}(t_A) = \frac{\alpha}{\alpha+\nu}(e^{-ft_A} - e^{-(f+\alpha+\nu)t_A}) \qquad \text{(S3.2)}$$

Where molecular diagnostics have not been used, an additional inoculation period, denoted by t_I hours, is given to the vectors, and hence the acquisition response curve is the proportion of plants that become infected after a fixed inoculation period. Hence, acquisition success without

testing is a combination of the distribution in Eq. S3.1 and the probability of inoculation in the fixed time $t_I = \tau$ (c.f. Eq. S2.10), i.e.,

Acquisition success, no testing

$$P_{AAP}(t_A, \tau) = \sum_{k=0}^{X_0} \binom{X_0}{k} p(t_A)^k (1 - p(t_A))^{X_0 - k} \left(1 - \left(\frac{\nu + f + \beta e^{-(\beta+\nu+f)\tau}}{\beta + \nu + f}\right)^k\right) \tag{S3.3}$$

Note that in the case of inoculation success, the form is the same whether or not molecular testing of insects is available, since a fixed period for insects to acquire the pathogen is always required prior to an inoculation period.

In other words, all IAP assays must be preceeded by a fixed acquisition period, $t_A = \tau$, in order to produce virus-bearing vectors. Therefore, the inoculation response curve is the proportion of plants that become infected after the variable inoculation period, i.e., a combination of the distribution in Eq. S1.12 and the probability of inoculation in the variable time t_I (c.f. Eq. S2.10):

Inoculation success $$P_{IAP}(\tau, t_I) = \sum_{k=0}^{X_0} \binom{X_0}{k} p(\tau)^k (1 - p(\tau))^{X_0 - k} \left(1 - \left(\frac{\nu + f + \beta e^{-(\beta+\nu+f)t_I}}{\beta + \nu + f}\right)^k\right) \tag{S3.4}$$

It is useful at this point to note simple general forms for the time dependence of acquisition and inoculation success when there is no testing. We do this by reference to the expressions for acquisition and inoculation success. For instance using Eq S3.4:

$$P_{IAP}(t_I) = 1 - (1-p(\tau))^{X_0} \sum_{k=0}^{X_0} \binom{X_0}{k} \left(\frac{p(\tau)}{1-p(\tau)} \frac{\nu + f + \beta e^{-(\beta+\nu+f)t_I}}{\beta+\nu+f} \right)^k$$

which using binomial expansion leads to,

$$\textit{Inoculation success, general form of time dependence} \quad f_{iap}(t) = 1 - (1 - D_1(1 - e^{D_2 t}))^{X_0} \quad . \tag{S3.5}$$

where X_0 is the initial number of insects (assumed known), and where D_1 and D_2 represent unknown constants. In the case of acquisition success, using Eq S3.3,

$$P_{AAP}(t_A, \tau) = 1 - (1-p(t_A))^{X_0} \sum_{k=0}^{X_0} \binom{X_0}{k} \left(\frac{c_1 p(t_A)}{1-p(t_A)} \right)^k$$

which using binomial expansion, and recalling the definition of $p(t)$, simplifies to,

$$\textit{Acquisition success, general form of time dependence} \quad f_{aap}(t) = 1 - \left(1 - C_1(e^{C_2 t} - e^{C_3 t})\right)^{X_0} \tag{S3.6}$$

where, as with inoculation success, X_0 is the initial number of insects (assumed known), and C_1, C_2 and C_3 represent unknown constants.
