# Supplementary material for: Estimating epidemiological parameters from experiments in vector access to host plants, the method of matching gradients
Source: PLoS Comput Biol. 2020 Mar 16;16(3):e1007724. doi: 10.1371/journal.pcbi.1007724 (PMC7098647; doi:10.1371/journal.pcbi.1007724)
Supplement: S4 Appendix — (PDF) [file pcbi.1007724.s004.pdf]

S4 Appendix, Method of matching gradients, deriving formulae for the gradients of response curves

Here we outline the calculation of initial acceleration and initial slope of acceleration for AAP and IAP experiments. This requires differentiating the solutions $P_{AAP}(t_A, \tau)$ and $P_{IAP}(\tau, t_I)$ (Eq.s S3.3 and S3.4) and evaluating at $t_A = 0$, and $t_I = 0$ respectively. In addition, we will derive the asymptote of $P_{IAP}(\tau, t_I)$. Note that we do not attempt to derive the asymptote of $P_{AAP}(t_A, \tau)$ as it equals zero when $f > 0$ (to see this notice that the longer the acquisition period the fewer the insects that survive).

AAP equation

Recalling that the full AAP solution is:

$$P_{AAP}(t_A, \tau) = \sum_{k=0}^{X_0} \binom{X_0}{k} p(t_A)^k (1 - p(t_A))^{X_0 - k} \left(1 - \left(\frac{\nu + f + \beta e^{-(\beta+\nu+f)\tau}}{\beta + \nu + f}\right)^k\right) \tag{S4.1}$$

where $p(t_A) = \frac{\alpha}{\alpha+\nu}(e^{-ft_A} - e^{-(f+\alpha+\nu)t_A})$ (see S1 Appendix). We differentiate the above system to find,

$$\begin{aligned}\frac{dP_{AAP}}{dt_A} = \sum_{k=0}^{X_0} \Bigg(&\binom{X_0}{k} k \left(\frac{p(t_A)}{1 - p(t_A)}\right)^{k-1} (1 - p(t_A))^{X_0} (1 - \theta^k) \frac{d}{dt_A}\left(\frac{p(t_A)}{1 - p(t_A)}\right) \\ &+ \binom{X_0}{k} \left(\frac{p(t_A)}{1 - p(t_A)}\right)^k X_0 (1 - p(t_A))^{X_0 - 1} (1 - \theta^k) \frac{d}{dt_A}(1 - p(t_A))\Bigg)\end{aligned} \tag{S4.2}$$

where $\theta = (\nu + f + \beta e^{-(\beta+\nu+f)\tau})/(\beta + \nu + f)$. We differentiate the above system again to find

$$
\begin{aligned}
\frac{d^2P_{AAP}}{dt_A^2} = \sum_{k=0}^{X_0}\binom{X_0}{k}\Bigg(& k(k-1)\Big(\frac{p(t_A)}{1-p(t_A)}\Big)^{k-2}(1-p(t_A))^{X_0}(1-\theta^k)\Big(\frac{d}{dt_A}\Big(\frac{p(t_A)}{1-p(t_A)}\Big)\Big)^2 \\
& + k\Big(\frac{p(t_A)}{1-p(t_A)}\Big)^{k-1} X_0(1-p(t_A))^{X_0-1}(1-\theta^k)\Big(\frac{d}{dt_A}(1-p(t_A))\Big)\frac{d}{dt_A}\Big(\frac{p(t_A)}{1-p(t_A)}\Big) \\
& + k\Big(\frac{p(t_A)}{1-p(t_A)}\Big)^{k-1}(1-p(t_A))^{X_0}(1-\theta^k)\frac{d^2}{dt_A^2}\Big(\frac{p(t_A)}{1-p(t_A)}\Big) \\
& + k\Big(\frac{p(t_A)}{1-p(t_A)}\Big)^{k-1} X_0(1-p(t_A))^{X_0-1}(1-\theta^k)\Big(\frac{d}{dt_A}(1-p(t_A))\Big)\frac{d}{dt_A}\Big(\frac{p(t_A)}{1-p(t_A)}\Big) \\
& + \Big(\frac{p(t_A)}{1-p(t_A)}\Big)^{k} X_0(X_0-1)(1-p(t_A))^{X_0-2}(1-\theta^k)\Big(\frac{d}{dt_A}(1-p(t_A))\Big)^2 \\
& + \Big(\frac{p(t_A)}{1-p(t_A)}\Big)^{k} X_0(1-p(t_A))^{X_0-1}(1-\theta^k)\Big(\frac{d^2}{dt_A^2}(1-p(t_A))\Big)\Bigg)
\end{aligned}
\tag{S4.3}
$$

The strategy for evaluating both $\frac{dP_{AAP}}{dt_A}\Big|_{t_A=0}$ and $\frac{d^2P_{AAP}}{dt_A^2}\Big|_{t_A=0}$ (i.e., Eqs S4.2 and S4.3 evaluated at $t_A = 0$), is to consider all the possible values of k in Eqs S4.2 and S4.3 and to retain only the terms that are non-zero.

For example, since $p(0) = 0$, the first term in Eq S4.2 can only be non-zero when $k = 1$ (i.e., for then $(\frac{p(t_A)}{1-p(t_A)})^{k-1} = 1$, which is zero for all other values of k). Similarly the second term in Eq S4.2 can only be non-zero when $k = 0$. Therefore,

$$
\frac{dP_{AAP}}{dt_A}\Big|_{t_A=0} = \binom{X_0}{1}(1-\theta)\frac{d}{dt_A}\Big(\frac{p(t_A)}{1-p(t_A)}\Big) + \binom{X_0}{0}X_0(1-p(t_A))^{X_0-1}(1-\theta^0)\frac{d}{dt_A}(1-p(t_A))
$$

Since the second term in the above equation is zero, the intial gradient for the acquisition response curve is,

$$\frac{dP_{AAP}}{dt_A}\Big|_{t_A=0} = X_0\alpha(1-\theta) \tag{S4.4}$$

where we have used the fact that $\frac{d}{dt_A}(\frac{p(t_A)}{1-p(t_A)}) = \alpha$, which follows from the expression for $p(t_A)$. Note that Eq. S4.4 appears as the entry in row 2 column 1 of Table 1, main text. Following the same strategy, initial acceleration for the acquisition response curve is found to be,

$$\frac{d^2P_{AAP}}{dt_A^2}\Big|_{t_A=0} = -X_0\alpha(1-\theta)\Big((X_0-1)\alpha(1-\theta)+2f+\nu+\alpha\Big) \tag{S4.5}$$

where we have used the fact that $\frac{d^2}{dt_A^2}(\frac{p(t_A)}{1-p(t_A)}) = \alpha(\alpha-2f-\nu)$ which follows from the expression for $p(t_A)$, so that,

$$\frac{d^2P_{AAP}}{dt_A^2}\Big|_{t_A=0}\Big(-\frac{dP_{AAP}}{dt_A}\Big|_{t_A=0}\Big)^{-1} - \Big(\frac{X_0-1}{X_0}\Big)\frac{dP_{AAP}}{dt_A}\Big|_{t_A=0} = 2f+\nu+\alpha \tag{S4.6}$$

Note that Eq. S4.6 appears as the entry in row 2 column 2 of Table 1, main text.

IAP equation

Recalling that the full IAP solution is:

$$P_{IAP}(\tau,t_I) = \sum_{k=0}^{X_0}\binom{X_0}{k}p(\tau)^k(1-p(\tau))^{X_0-k}\left(1-\left(\frac{\nu+f+\beta e^{-(\beta+\nu+f)t_I}}{\beta+\nu+f}\right)^k\right), \tag{S4.7}$$

we differentiate the above system to find,

$$\frac{dP_{IAP}}{dt_I} = \sum_{k=0}^{X_0} \binom{X_0}{k} p(\tau)^k (1-p(\tau))^{X_0-k} \left(k \left(\frac{\nu + f + \beta e^{-(\beta+\nu+f)t_I}}{\beta+\nu+f} \right)^{k-1} \beta e^{-(\beta+\nu+f)t_I} \right), \tag{S4.8}$$

and again to find,

$$\frac{d^2P_{IAP}}{dt_I^2} = \sum_{k=0}^{X_0} \binom{X_0}{k} p(\tau)^k (1-p(\tau))^{X_0-k} \Big(-k(k-1) \left(\frac{\nu + f + \beta e^{-(\beta+\nu+f)t_I}}{\beta+\nu+f} \right)^{k-2} * \\ (\beta e^{-(\beta+\nu+f)t_I})^2 - k\beta(\beta+\nu+f) \left(\frac{\nu + f + \beta e^{-(\beta+\nu+f)t_I}}{\beta+\nu+f} \right)^{k-1} e^{-(\beta+\nu+f)t_I} \Big) \tag{S4.9}$$

Therefore, the first two initial gradients for the inoculation response curves are,

$$\frac{dP_{IAP}}{dt_I}|_{t_I=0} = \beta \langle y_0(\tau) \rangle \tag{S4.10}$$

$$\begin{aligned} \frac{d^2P_{IAP}}{dt_I^2}|_{t_I=0} &= -\beta\langle y_0(\tau)\rangle(\nu+f) - \beta^2 \sum_k k^2 \binom{X_0}{k} p(t_I)^{X_0-k}(1-p(t_I))^k \\ &= -\beta\langle y_0(\tau)\rangle(\nu+f) - \beta^2(\langle y_0(\tau)\rangle^2 + Var(y_0)) \\ &= -\beta\langle y_0(\tau)\rangle(\nu+f) - \beta^2(\langle y_0(\tau)\rangle^2 + \langle y_0(\tau)\rangle(1-p(\tau))) \end{aligned} \tag{S4.11}$$

where the final step makes use of the fact that $Var(y_0) = \langle y_0(\tau) \rangle (1 - p(\tau))$ since y_0 is binomially distributed. Finally, we find the asymptote of $P_{IAP}(\tau, t_I)$. Letting $t_I \to \infty$ in Eq. S4.7 we find that,

$$
\begin{aligned}
\lim_{t_I \to \infty} P_{IAP}(\tau, t_I) &= \sum_{k=0}^{X_0} \binom{X_0}{k} p(\tau)^k (1-p(\tau))^{X_0-k} \left(1 - \left(\frac{\nu+f}{\beta+\nu+f}\right)^k\right) \\
&= 1 - (1-p(\tau))^{X_0} \sum_{k=0}^{X_0} \binom{X_0}{k} \left(\frac{p(\tau)}{1-p(\tau)} \frac{\nu+f}{\beta+\nu+f}\right)^k \\
&= 1 - (1-p(\tau))^{X_0} \left(1 + \left(\frac{p(\tau)}{1-p(\tau)} \frac{\nu+f}{\beta+\nu+f}\right)\right)^{X_0} \\
&= 1 - \left((1-p(\tau)) + p(\tau)\frac{\nu+f}{\beta+\nu+f}\right)^{X_0} \\
&= 1 - \left(1 - \frac{\beta p(\tau)}{\beta+\nu+f}\right)^{X_0}
\end{aligned}
\tag{S4.12}
$$

Recalling from Eq. S4.10 that,

$$
\begin{aligned}
\frac{dP_{IAP}(t_I,\tau)}{dt_I}|_{t_I=0} &= \beta \langle y_0(\tau) \rangle \\
&= \beta X_0 p(\tau)
\end{aligned}
\tag{S4.13}
$$

where we have used the relation that $\langle y_0(\tau) \rangle = X_0 p(\tau)$, see Eq. S1.13. Note that Eq. S4.13 appears as the entry in row 3 column 1 of Table 1, main text. Finally, re-arranging Eq. S4.12 and substituting in Eq. S4.13 we see that,

$$
(\nu + f + \beta) = \frac{P'_{IAP}|_{t_I=0}}{X_0} \left(1 - \left(1 - \lim_{t_I \to \infty} P_{IAP}(\tau, t_I)\right)^{1/X0}\right)^{-1}
\tag{S4.14}
$$

which appears as the entry in row 3 column 2 of Table 1, main text.
