# Supplementary material for: Estimating epidemiological parameters from experiments in vector access to host plants, the method of matching gradients
Source: PLoS Comput Biol. 2020 Mar 16;16(3):e1007724. doi: 10.1371/journal.pcbi.1007724 (PMC7098647; doi:10.1371/journal.pcbi.1007724)
Supplement: S5 Appendix — (PDF) [file pcbi.1007724.s005.pdf]

S5 Appendix, Steps taken to estimate epidemiological parameters

The gradient derivations from S4 Appendix can be summarized as the following sequence of identities,

From Eq. S4.14:

$$\nu + f + \beta = \frac{P'_{IAP}|_{t_I=0}}{X_0}\left(1 - (1 - \lim_{t_I\to\infty} P_{IAP}(\tau, t_I))^{1/X0}\right)^{-1} \tag{S5.1}$$

From Eq. S4.6:

$$\alpha + \nu + 2f = \left(\frac{d^2P_{AAP}}{dt_A^2}\Big|_{t_A=0}\right)\left(-\frac{dP_{AAP}}{dt_A}\Big|_{t_A=0}\right)^{-1} - \frac{dP_{AAP}}{dt_A}\Big|_{t_A=0}\left(1 - \frac{1}{X_0}\right) \tag{S5.2}$$

From substituting the expression for θ into Eq. S4.4:

$$\alpha\beta = (\nu + f + \beta)(X_0)^{-1}\left(\frac{dP_{AAP}}{dt_A}\Big|_{t_A=0}\right)(1 - e^{-(\nu+f+\beta)\tau})^{-1} \tag{S5.3}$$

From Eq. S4.10 and Eq. S1.13:

$$\frac{\alpha\beta}{\alpha + \nu}(e^{-f\tau} - e^{-(f+\alpha+\nu)\tau}) = (X_0)^{-1}\frac{dP_{IAP}}{dt_I}\Big|_{t_I=0} \tag{S5.4}$$

which can be reduced to an equation in f by substituting identities S5.2 and S5.3 into S5.4. The resulting equation can be solved for an estimate of f (i.e., f^*). In this way the four equations in

four unknowns can be combined to produce parameter estimates. There are many ways that f^* in turn can be combined with the identities to produce the remaining parameter estimates (i.e., α^*, β^* and ν^*). The following are the steps that we have taken.

Combining f^* with identity S5.1 produces an expression for $(\nu + \beta)^*$. Combining f^* with identity S5.2 produces an expression for $(\nu + \alpha)^*$. Combining these two terms produces an expression for $(\beta - \alpha)^*$. We then solved the quadratic equation $x^2 - x(\beta - \alpha)^* - (\beta\alpha)^* = 0$ which has the solutions $x = \beta^*, -\alpha^*$. Note that the final term of the quadratic equation is the right hand side of the identity S5.3 combined with S5.1. Finally, $\nu^* = (\nu + \alpha)^* - \alpha^*$.
