# Supplementary material for: Estimating epidemiological parameters from experiments in vector access to host plants, the method of matching gradients
Source: PLoS Comput Biol. 2020 Mar 16;16(3):e1007724. doi: 10.1371/journal.pcbi.1007724 (PMC7098647; doi:10.1371/journal.pcbi.1007724)
Supplement: S6 Appendix — (PDF) [file pcbi.1007724.s006.pdf]

# S6 Appendix, Data appearing in the access period experiment of Maruthi et al. (2016)

In this section we have reproduced data reported in Maruthi *et al.* (1) as a series of three tables. First, in Table A the acquisition and inoculation assay results (Table 3 in (1)) are presented. We used this data to infer the CBSV epidemiological parameter estimates, Table 3, main text. Note that the data in this table were also compared with the results of dynamic simulation based on the CBSV parameter estimates (first portion of our dynamic simulation results, Fig. 4A-B, main text).

Next, in Table B the results of an experiment in which the number of whiteflies is varied are presented. We compared this data with the results of dynamic simulation based on the CBSV parameter estimates (second portion of our dynamic simulation results, Fig. 4C, main text).

Finally, in Table C the results of an experiment featuring a period of feeding intermediate to acquisition period and inoculation period are presented. We compared this data with the results of dynamic simulation based on the CBSV parameter estimates (third portion of our dynamic simulation results, Fig. 5, main text).

Table A: **Data reproduced from Table 3 in (1) and used in this paper to infer parameter estimates**

| [a] | **Determining AAP for CBSV on cassava**[b] | | **Determining IAP for CBSV on cassava**[c] | |
|---|---|---|---|---|
| **Time period** | **No. of plants infected/inoculated** | **% transmission achieved** | **No. of plants infected/inoculated** | **% transmission achieved** |
| 0 | 0 | 0 | 0 | 0 |
| 5–10 min | 4/25 | 16.0 | 6/31 | 19.3 |
| 30 min | 8/25 | 32.0 | 7/33 | 21.2 |
| 1 hr | 10/25 | 40.0 | 8/39 | 20.5 |
| 4 hr | 6/15 | 40.0 | 13/35 | 37.1 |
| 24 hr | 9/20 | 45.0 | 29/48 | 60.4 |
| 48 hr | 6/15 | 40.0 | 6/15 | 40.0 |

Table notes: We added the additional row for ' Time period'= 0 that was not included in (1) (since the 'zero row' is trivially true). Table 3 in (1) includes the following footnotes: [a] 'About 20–25 viruliferous whiteflies inoculated each plant in this experiment'; [b] 'Suspected viruliferous whiteflies were given a standard 48 hr inoculation access period (IAP) for testing different acquisition access periods (AAPs)'; [c] 'Suspected viruliferous whiteflies were given a standard 48 hr acquisition access period (AAP) for testing different inoculation access periods (IAPs)'. In accordance with [a], we used a value of 22.5 whiteflies for inference of the epidemiological parameter estimates. This was rounded to 23 for the dynamic simulations (since integer values are required). In accordance with [b] and [c] both inference of parameter estimates and dynamic simulation of the AAP and IAP assays were conducted with the fixed access period value $\tau = 48$ hr.

Table B: **Data reproduced from Table 1 in (1) and used in this paper for comparison with dynamic simulation**

| **No. of whiteflies used to inoculate each plant** | **AAP** | **IAP** | **No. of plants infected/inoculated** | **% transmission achieved** |
|---|---|---|---|---|
| 20–25 | 4 days | 5 days | 7/20 | 30.0 |
| 50–100 | 4 days | 5 days | 14/26 | 53.0 |

Table notes: An additional row for 'Whiteflies emerging from CBSD-affected cassava plants' was not relevant to our study and has been omitted. Row 1 and 2 present range of whiteflies used in the experiment, for our dynamic simulations of the affect of whitefly density we took $X_0 = 20$ (row 1) and $X_0 = 50$ (row 2) as our parameter value.

Table C: **Data reproduced from Table 2 in (1) and used in this paper for comparison with dynamic simulation**

| **Mode of transmission tested** | **No. of whiteflies per plant** | **AAP** | **IAP** | **No. of plants infected/inoculated** | **% transmission achieved** |
|---|---|---|---|---|---|
| Persistent mode of transmission | 10–20 | 24 hr | 48 hr+48 hr | 0/15 | 0 |
| | 7–20 | 48 hr | 48 hr+48 hr | 0/15 | 0 |

Table notes: Additional rows for 'Non-persistent mode of transmission' and 'Semipersistent mode of transmission' were not relevant to our study and have been omitted. While Table 2 in (1) suggests that an intermediate period of 48 hr was used, elsewhere (Results and Discussion sections) the authors discuss an intermediate period of 24 hr and 48 hr. For comprehensiveness we present results in this study for both 24 hr and 48 hr intermediate periods.

# References

[1] Maruthi MN, Jeremiah SC, Mohammed IU, Legg JP. The role of the whitefly, Bemisia tabaci (Gennadius), and farmer practices in the spread of cassava brown streak ipomoviruses. J Phytopathol. 2016; 31: 1–11.
